# Phenolic Profiling and Evaluation of Contraceptive Effect of the Ethanolic Extract of *Salsola imbricata* Forssk. in Male Albino Rats

**DOI:** 10.1155/2014/695291

**Published:** 2014-12-21

**Authors:** Naglaa Gamil Shehab, Eman Abu-Gharbieh

**Affiliations:** ^1^Department of Pharmacognosy, Faculty of Pharmacy, Cairo University, Giza, Egypt; ^2^Department of Pharmacology and Toxicology, Dubai Pharmacy College, P.O. Box 19099, Dubai, UAE

## Abstract

Reported researches dealing with either composition or bioactivity of *Salsola imbricata* are limited. This study was conducted aiming to investigate the phenolic composition of the plant and evaluate its efficacy as male contraceptive. Polyphenols, namely, phenolic acids and flavonoids, were qualitatively and quantitatively analysed by RP-HPLC in the hydrolysed methanol extract using two different wavelengths, 280 and 330 nm. The efficiency of different solvents in extracting the plant phenolics was assessed via spectrophotometric determination of the total phenolic and flavonoid contents. Acute toxicity study was carried out on the ethanolic extract to ascertain its safety prior to biological evaluation. The contraceptive effect was assessed, in male rats, by oral administration of the extract at two doses (250 and 500 mg/kg b. wt.), over a period of 65 days. HPLC analyses allowed the identification and quantification of a total of 13 and 8 components in the hydrolysed-methanol extract; the overall phenolic composition was dominated by quercitrin (12.692%) followed by coumaric acid (4.251%). Prolonged oral administration of the ethanolic extract caused slight reduction in the testis weight only. A significant decrease in the sperm count was observed (*P* < 0.01) in the two treated groups while significant decrease in the epididymal sperm motility was only observed in the high dose group. Morphological abnormalities were observed in sperms of treated animals. No distinct change in serum FSH, LH, and testosterone concentration was recorded. The histopathological findings supported to a high extent these results. The male contraceptive activity of *Salsola imbricata* could be ascribed to its phenolic components, especially quercitrin.

## 1. Introduction

Contraceptive methods are usually classified as either modern or traditional. Compared with female contraceptives, male alternatives are few and relatively underused. Traditional male contraceptive methods include periodic abstinence and withdrawal; meanwhile, the modern ones account for only 8.9% of global contraceptive use and involve the application of condom (barrier method) or vasectomy (sterilization) [1].

Despite the fact that there is no single new male contraceptive drug brought to the market, experimental trials are continuously done on large number of chemicals to introduce hormonal or pharmacologic agents. However, the results obtained were in most cases unsatisfactory due to side effects, incomplete efficacy, or irreversibility resulting from total spermatogenic arrest [2]. The search for an effective, safe, and reversible male antifertility (contraceptive) drug with minimum side effects remains thus a challenge.

Plants have been always praised for their potential health benefits mainly in folk medicine. They are now highly investigated as source of safe phytopharmaceuticals. In this respect, the antifertility activity of several plant extracts has been evaluated in both male and female. Some of these exerted spermicidal effects; some caused a reduction in sperm counts and altered sperm mobility [3], and others produced testicular changes and altered hormonal levels [4]. In addition, a noticeable antifertility potential was recorded for a number of plant metabolites; among these are alkaloids, saponins, steroids, phenolic acids, and flavonoids [5–8]. Particularly, gossypol (a phenolic derived from the cotton plant) is second to hormonal contraception in the volume of published literature including phase 3 trials and is believed to work by inhibiting spermatogenesis and sperm motility [9, 10].

Plants of genus* Salsola* (Chenopodiaceae or Amaranthaceae) grow in most arid places of the world, along sea shores, in grassland, and desert communities [11] and are commonly utilized as traditional medicines. The aqueous extract of* Salsola tuberculatiformis*, used as oral contraceptive by Bushmen women in Southern Africa, was found to produce prolonged gestation in sheep and contraception in female rats [12].

*Salsola imbricata* Forssk. (Arabic names: Harm, Al-ghtraf, Khareet Chaundhary, el-Chrêt, and Arkam) is a shrub wild growing in Middle East deserts; it is distributed throughout Central and Southwest Asia, North Africa, and Mediterranean countries [13, 14].* Salsola imbricata* is used by the local people in UAE for contraceptional purposes [15]. The plant has several other folk medicinal applications, especially in Saudi Arabia, where it is used as anthelmintic and snuff for cleaning sinuses and for alleviating dermal itching [15]. It is also traditionally used as insecticide, cathartic, and diuretic [16].

Previous phytochemical investigations of the plant were limited. A chromatographic analysis of the alcoholic extract from* Salsola imbricata* yielded two new secondary metabolites, salisomide and salisoflavan; their structures were established with the help of spectroscopic techniques including COSY, HMQC, and HMBC NMR experiments [17]. Later on, two triterpenoidal saponin glycosides were isolated and identified from the roots of the Egyptian plant [18]. Apart from these data, scientifically based information on either composition or bioactivities of the plant was negligible, thus stimulating the performance of further research.

The current study focused on evaluation of the male antifertility potential of the alcoholic extract of* Salsola imbricata*, guided by its folk use as contraceptive, and investigation of its composition especially the phenolic content.

## 2. Materials and Methods

### 2.1. Chemicals

Ethanol, methanol, acetone, and ethyl acetate were purchased from Fisher Scientific, USA, while Folin-Ciocalteu reagent, gallic acid, NaNO_2_, aluminum chloride, sodium hydroxide, and carboxymethyl cellulose were purchased from Merck, Darmstadt, Germany.

### 2.2. Plant Material

*Salsola imbricata* Forssk. plants were collected during September (2012) from Muhaisnah Desert, Dubai, UAE. The plant was kindly identified by Dr. Hassnaa Ahmed Hosny, Professor of Plant Taxonomy, Department of Botany, Faculty of Science, Cairo University, Egypt. Voucher specimens were kept at the Herbarium of the Pharmacognosy Department, Faculty of Pharmacy, Cairo University. Whole plants, air-dried in shade, were powdered and preserved for further study.

### 2.3. Extraction and Phytochemical Screening

The air-dried whole plant powder of* Salsola imbricata* (3.3 kg) was exhaustively extracted by cold maceration in 70% ethanol (5 L × 4). The solvent was evaporated under reduced pressure at 50°C to provide a greenish-brown residue (*Salsola imbricata* dried ethanolic extract, 148.42 g). Portions of both powder and extract were subjected to phytochemical screening through specific chemical tests and TLC for qualitative identification of its components [19, 20].

### 2.4. HPLC Profiling of the Phenolic Composition

The phenolic composition was investigated in aliquots (1 g, each) of the hydrolysed-methanolic extract of the plant via RP-HPLC on a Hewlett Packard HPLC System (HP 1050 HPLC DAD w/Data System). Analyses were carried out at operating conditions suitable for detection of either phenolic acids or flavonoids [21, 22].

For phenolic acids, the apparatus was equipped with an Alltima C18 column (particle size 5 mm, 150 × 4.6 mm) and Alltima C18 guard column (5 mm) (Alltech, USA), the UV detector being set at 280 nm. Meanwhile, the separation of flavonoids was carried out on a Hypersil-ODS C18 column (particle size 5 *µ*m, 4.6 × 250 mm) and the UV detector was set at 330 nm. All analyses were performed at 35°C; gradient elution was employed using acetonitrile-acetic acid mixtures as mobile phase, at a flow rate of 1 mL/min, and the injected volume was 10 *µ*L for both standard and tested samples. Authentic reference samples were prepared by diluting stock solutions with methanol to afford a final concentration of 50 *µ*g/mL. Identification of the individual components was performed by comparing their retention times with those of the available standards similarly analyzed. Quantification was based on peak area computation using the external standard method. All analyses were carried out in triplicate. Results are recorded in Tables 1 and 2 for samples analysed at 280 and 330 nm, respectively.

### 2.5. Influence of Extracting Solvent on Total Phenolic and Flavonoid Contents

Solvents of different polarities, namely, 70% ethanol and methanol, acetone, and ethyl acetate, were individually used for extraction of the air-dried powdered material (100 g, each). The efficiency of the extracting solvent was monitored by colorimetric estimation of total phenolic and flavonoid contents using a spectrophotometer (UV-1700 PharmaSpec, Shimadzu, Japan). All experiments were carried out in triplicate.

The* total phenolic content* was determined by using Folin-Ciocalteu reagent (Merck, Darmstadt, Germany) as described by Singleton and Rossi [23] and modified by Oktay et al. [24]. Results were expressed as mg/g gallic acid equivalent, calculated on dry weight of the plant material; serial dilutions of gallic acid (10, 20, 30, 40, and 50 *µ*g/mL) were used for establishment of the calibration curve. Aliquots (1 mL, each) of the tested samples and standard were, separately, added to a volumetric flask containing 9 mL of water followed by addition of 1 mL of Folin-Ciocalteu reagent and the reaction mixture was carefully blended by vortex. After 5 min, 10 mL of 7% sodium carbonate was added to the mixture which was further incubated for 90 minutes, at room temperature. Finally, the absorbance was determined at 750 nm against the reagent blank.

The* total flavonoid content* of the prepared extracts was measured, spectrophotometrically, by the aluminum chloride method and quercetin as a standard following the procedure described by Dewanto et al. [25]. The plant extracts (0.1 mL, each) were added to 0.3 mL distilled water followed by 5% NaNO_2_ (0.03 mL) and the reaction mixture was left for 5 min, at 25°C. Aluminum chloride (0.03 mL, 10%) was then added and the mixture was left for another 5 min, then treated with 0.2 mL of 1 mM NaOH, and finally diluted to 1 mL with water and the absorbance of the yellow colour produced read at 510 nm.

### 2.6. Experimental Animals

All antifertility experiments were performed on sexually matured Sprague Dawley rats of both sexes weighing 200–220 g. Male and female albino mice (weight, 25 ± 5 g) were used for acute toxicity study. Animals were kept under the same standard hygienic conditions (temperature 22 ± 2°C, relative humidity 50–60%, with 12 h day/night lighting cycle) and fed with well-balanced normal diet and water supplied ad libitum. They were left for a period of one week for accommodation before performing the experiments. All animals' investigations were performed in accordance with the ethical standards for the proper care and use of laboratory animals and upon approval of the Research and Ethical Committee of the Dubai Pharmacy College, Dubai, UAE.

### 2.7. Acute Toxicity Study

The study was conducted in albino mice of both sexes. The ethanolic extract was administered orally at different doses (250, 500, 1000, 2500, and 5000 mg/kg). Mortality and manifestation of toxicity were recorded during 72 h. Based on the mortality rate, probit values, the oral LD_50_ was determined as described by McLeod [26].

### 2.8. Treatment Protocol

Three groups of matured male rats (6 rats per group) were selected for this experiment and treated orally as follows: group I received 1% carboxymethyl cellulose (CMC) as vehicle control while group II and group III were administrated the ethanolic extract of* Salsola imbricata* at doses of 250, 500 mg/kg body weight, respectively (5 and 10% of the LD_50_), on consecutive days for 65 days. Twenty-four hours after their last dose and 18 h of fasting, the rats were sacrificed under light ether anesthesia.

Blood was obtained by cardiac puncture from the animals for hormonal analysis. The testis, cauda epididymal ducts, seminal vesicles, and the prostate gland were dissected out and cleaned from adherent fats and their weights were recorded.

### 2.9. Fertility Test

The fertility index was determined by Oberlander et al. Male rats were introduced to parous females (male : female ratio, 1 : 2) in the evening for six consecutive days starting from day 60. The successful mating was confirmed by the presence of sperms in the vaginal smears examined on the next morning from 61 to 66 days and indicated that the females had mated with the particular male and the day of mating was taken to be day 1 of pregnancy. Fertility test was considered positive when implantation sites were present and fertility index was calculated according to the following equation [27]:

(1) $$\begin{array}{c} \text{Fertility}\;\;\text{index}=100\times \frac{\text{number}\;\;\text{pregnant}\;\text{animals}}{\text{number}\;\;\text{paired}\;\text{animals}}. \end{array}$$

### 2.10. Body and Organ Weights

The initial and final animals body weights were recorded. Additionally, the testes, epididymides, seminal vesicle, and ventral prostate were weighed to the nearest milligram and the results are presented in Table 3.

### 2.11. Sperm Count

Cauda epididymal sperm count was performed according to WHO [28]. 100 mg of cauda epididymides was minced into 1 mL of normal saline. One drop of evenly mixed suspension was applied on the chamber and covered with a cover-glass. The sperm heads within the squares of the grid were counted; the number of sperms was counted in a strip of 10 squares. This procedure was repeated five times for every sample to get accurate readings and the average was determined. The sperm count was calculated in millions per milliliter of sperm suspension as follows:

(2) $$\begin{array}{c} \text{Sperms}\;\;\text{count}=\frac{\text{Number}\;\text{of}\;\text{sperms}}{10\;\;\text{squares}}\times \text{dilution}\;\text{factor}. \end{array}$$

### 2.12. Sperm Motility

The percent motility was determined by counting both motile and immotile spermatozoa per unit area [29]. Accurate half centimeter from vas deferens, from both control and treated animals, was flushed with 1 mL of Tyrode's solution kept previously at 37°C. A drop from this sperm solution was put in the center of the lower disc of the Makler chamber and carefully covered with a cover-glass. Both nonmotile and the motile sperms were counted within 10 squares in the same areas of the grid. The procedure was repeated in quadruplicate for each sample studied and the mean was determined.

### 2.13. Epididymal Sperm Morphology

A drop of diluted semine was transferred to an Eppendorf tube containing one drop of 10% nigrosin and one drop of 1% eosin. The viability test for the sperms was done and the total sperm abnormality was expressed as percentage incidence according to the method described in the WHO laboratory manual [30]. The results are recorded in Table 4.

### 2.14. Radioimmunoassay of Hormones

Blood samples were collected for estimations of serum gonadotropins and testosterone by radioimmunoassay (RIA). Serum samples were separated by standard procedures and stored at −20°C for subsequent analysis and the concentrations of luteinizing hormone (LH), follicle stimulating hormone (FSH), and testosterone were measured in duplicate. The results are shown in Table 5.

### 2.15. Testicular Histology

The right testis of each animal was fixed in Bouin's fluid, dehydrated in graded ethanol, cleared in xylene, and embedded in paraffin wax. Sections were cut at 5 mm, stained with Harris' hematoxylin and eosin, and observed under a light microscope.

### 2.16. Statistical Analysis

The results are presented as mean ± SME and analyzed by one-way ANOVA followed by Bonferroni's post tests using GraphPad Prism version 6. Values were considered significant at *P* < 0.05.

## 3. Results

### 3.1. Phytochemical Screening

Screening of powdered* Salsola imbricata*, by means of chemical tests, revealed the presence of carbohydrates and/or glycosides, flavonoids, tannins, sterols and/or triterpenes, and saponins while alkaloids could not be detected. Furthermore, TLC investigation of the 70% ethanolic extract confirmed the occurrence of steroids, triterpenoids, and polyphenols.

### 3.2. HPLC Profiling of Phenolics

RP-HPLC analyses of the hydrolyzed-methanolic extract allowed the identification and quantitation of several phenolics. A total of 13 components (corresponding to 13.904% of the total composition) were identified at 280 nm (Table 1), among which 9 were phenolic acids (9.734%) with prevalence of coumaric acid (4.251%) and 2 flavonoids (catechin and chrysin) besides the diphenol, catechol; meanwhile, only one (benzoic acid, 2.306%) was nonphenolic. On the other hand, by setting the detector at *λ* = 330 nm, 8 components were identified (Table 2) among which 7 were of flavonoidal nature with major quercitrin (12.692%); besides, rosmarinic acid (detected in relatively appreciable amount, 2.734%) was the only phenolic acid.

### 3.3. Influence of Extracting Solvent on Total Phenolic and Flavonoid Contents

Spectrophotometric evaluation of the total phenolic content of the different extracts of* Salsola imbricata* revealed the highest yield in that of acetone (amount of total phenolics 4 mg of gallic acid equivalents (mg GAE)/g of plant dry weight) followed by methanol, ethyl acetate, and 70% ethanol (2.6, 0.93, and 0.64 mg GAE/g, resp.).

On the other hand, the total flavonoid content expressed as quercetin g/100 g dry plant material was observed in the methanol extract (0.571%) followed by those of acetone, 70% ethanol, and ethyl acetate extracts (0.374, 0.217, and 0.11%, resp.).

### 3.4. Acute Toxicity Study

The ethanolic extract of* Salsola imbricata* was found to be safe up to dose 5 g/kg. There were no signs of morbidity or behavioral changes in any of the treated groups of animals during the period of observation. The wide safety margin of the ethanolic extract of* S. imbricata* is highly encouraging the biological evaluation.

### 3.5. Antifertility Study

#### 3.5.1. Fertility

The number of fertile males decreased in all treated groups as 50% of the animals in group II were infertile while all animals in group III were totally infertile. The ratios between delivered and inseminated females in groups II and III were 3/6, 0/6 versus 6/6 animals in group I, respectively. The number of pups in group II was 24 versus 73 pups in group I dropped after prolonged administration of the ethanolic extract. All delivered pups were normal and healthy.

#### 3.5.2. Body and Organs Weights

Prolonged oral administration of the ethanolic extract of* Salsola imbricata* at doses of 250 and 500 mg/kg to the male rats for 65 days caused a slight decrease in the body weight, weight of the testis and epididymis but actually those reductions were statistically not significant and both groups showed no significant changes in the weight of the other sexual organs comparing to the control group as shown in Table 3.

#### 3.5.3. Sperms Count

Significant change in the sperms count was observed in the treated groups compared to the controlled animals in a dose dependent manner (*P* < 0.01) as shown in Table 4.

#### 3.5.4. Sperm Motility and Morphology

The epididymal sperm motility was decreased in both treated groups. However, this reduction was statistically significant only in group III that has been treated with the highest dose (*P* < 0.01) compared with that of the control group. A significant increase in the percentage of the sperm abnormalities was noticed in both treated groups as shown in Table 4. These abnormalities were found to be more in the tail represented by bent and/or kinked tails.

#### 3.5.5. Radioimmunoassay of Hormones

No significant change in the levels of serum testosterone, FSH, and LH was observed compared to control animals (*P* > 0.05) as shown in Table 5.

#### 3.5.6. Testicular Histology

**Figure 1:**
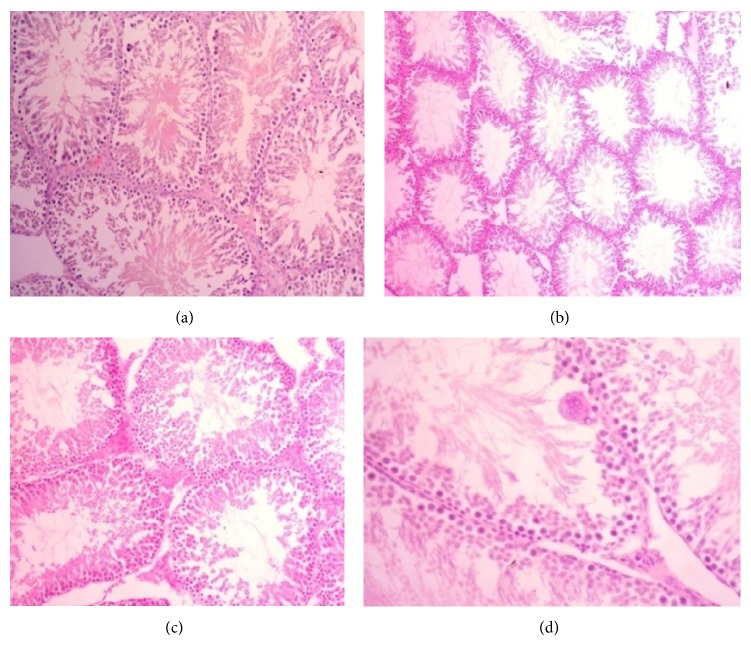
(a) Testis of a rat of group I (vehicle treated control) showing normal features with successive stages of transformation spermatid of seminiferous epithelium to spermatozoa; H&E, ×200. (b) Testis of rat of group II (25 mg/kg* S. imbricata*) showing moderate reduction of mature spermatid; H&E, ×200. (c) Testis of rat of group III (500 mg/kg* S. imbricata*) showing significant reduction in mature spermatid; H&E, ×200. (d) Testis of rat of group III (500 mg/kg* S. imbricata*) revealing presence of giant cells in some tubules; H&E, ×400.

The testis of group I (vehicle treated control) animals showed normal features with successive stages of transformation of the seminiferous epithelium into spermatozoa. Leydig cells were situated in between the tubules (Figure 1(a)). Histopathological examination of the testis after 65 days of treatment showed a clear correlation between the dose and the lesion severity. In rats treated with 250 mg/kg, po (group II) produced diffuse changes of the tubules with reduction of mature spermatid (Figure 1(b)). In rats treated with 500 mg/kg, po (group III) almost all tubules were affected as represented by the presence of giant cells. A significant reduction in the mature spermatid was also observed (Figures 1(c) and 1(d)).

## 4. Discussion

A large number of scientists are searching for a relatively cheap, widely available, easily accepted, and effective contraceptive of plant origin that is equally noninvasive, nonhormonal in action, nontoxic, and relatively long acting. Medicinal plants are important elements of indigenous medical system in many countries. Nowadays, the use of traditional medicines has received considerable interest and a large number of plants have been screened for their antifertility activity [31–34]. Thus the present study was designed to investigate the effect of the ethanolic extract of* Salsola imbricata* on the fertility of male rats and to investigate the phenolic constituents which may be responsible for this activity. The wide distribution of this desert plant and its common use in folk medicine and as animal fodder stimulated also the performance of this investigation. In addition, screening of the ethanolic extract indicated the presence of steroids, triterpenoids, and polyphenols which could exert an antifertility effect [5–8].

A number of polyphenols were identified in the hydrolyzed-methanolic extract of* Salsola imbricata* including simple phenolics, phenolic acids, and flavonoids among which coumaric acid and quercitrin were prevalent. The best solvent for extraction of the plant phenolics was acetone followed by methanol; meanwhile, flavonoids were detected in the highest amount in methanol. However, for safety and economic considerations, the ethanol (70%) was selected for further biological study.

Obviously, the body and sexual organs weights of the treated rats were not affected on prolonged administration of this extract over a period of 65 days, at any dose level. However, a significant decrease in the epididymal sperm count could be observed in both treated groups; meanwhile, a significant reduction in sperms motility was only noticed in the high dose group III (500 mg/kg). In addition, a significant increase in sperm abnormalities was observed in both treated groups; this is more evident in the tail morphology as bent and/or kinked tails. In contrast, the* Salsola* extract did not affect any of the reproductive hormones levels.

The drop in the ratio between delivered and inseminated females was clear following 65 days of treatment, despite the insemination of all females. Thus treated males failed to impregnate females, and this was mainly observed at the high dose level (500 mg/kg). The histopathological findings supported to a high extent the pharmacological ones; since a significant reduction in spermatid maturation was evident in testis of the treated animals, this effect was reflected by the characteristics of the sperm movement, and the swimming trajectories from the treated group were irregular and nonprogressive; those characteristics are usually exhibited by immature spermatozoa [35].

Taking in consideration all the previous findings, the observed antifertility effect of* S. imbricata* on male rats could be attributed to its phenolic constituents, especially quercitrin. As a matter of fact, these metabolites have been proven to produce antiandrogenic activity and to affect fertility in both male dogs and albino rats [31, 36, 37]. The flavonol glycoside quercitrin was prevailing (12.692%) among phenolics detected by HPLC in the methanolic extract. In addition to antioxidant properties, a number of interesting activities have been attributed to quercetin aglycone and/or its glycosides quercitrin and rutin [38]. They are increasingly receiving interest as new generation of anticancer molecules and were recognized by multidrug resistant (MDR) transporters such as P-glycoprotein and MRP1 protein [39–41]. In a previous study, quercitrin was found to be able to inhibit the function of P-glycoprotein noncompetitively in living K562/adr and GLC4/adr cells [38]. Interestingly, multidrug resistance (MDR) assay revealed that rat epididymal cells and epididymal spermatozoa display an MDR phenotype that can be inhibited by alkylphenols [42]. Recently, the MDR transport activity was claimed to protect oocytes from chemotherapeutic agents and xenobiotics during their maturation [43]. This effect observed in oogenesis could probably be extended to the spermatogenesis process. Since extreme abnormalities in sperms shapes and motility were noticed in the treated groups, these effects could be related to the ability of quercitrin to inhibit P-glycoprotein noncompetitively, thus rendering the sperms less protected against xenobiotics and toxins during spermatogenesis. Moreover, our findings are in agreement with those reported by Kachhawa et al. [36] who confirmed the contraceptive potential of certain fractions of the methanolic extract of the stem of* Dendrophthoe falcata*, in male albino rats; the major constituent in* D. falcata* was identified as quercitrin. Furthermore, phenolic acids especially caffeic, coumaric, benzoic, and salicylic acids, which were also detected in the currently investigated extract, have well documented antifertility activities [32, 44–46] and may thus contribute to the antifertility effect of the plant.

## 5. Conclusions

Exploration of the antifertility potential of* Salsola imbricata* plant via prolonged oral administration of its ethanol extract to male rats revealed that antifertility effect was produced by the plant. This activity was related to its phenolic content. The ethanol extract could thus be recommended, after extensive laboratory and clinical trials, as reversible herbal male contraceptive, especially that it showed a high safety margin. To the best of our knowledge, this is the first report on the antifertility effect and phenolic profiling of* Salsola imbricata.*

## Figures and Tables

**Table 1:** Phenolics identified by RP-HPLC analysis (at *λ* = 280 nm) of the methanolic extract of *Salsola imbricata*.

Retention time	Identified constituent	Relative area %
6.89	Gallic acid	0.145
8.177	Protocatechuic acid	0.068
8.396	Catechin	0.461
8.653	Chlorogenic acid	0.377
8.967	Catechol	0.329
9.949	Caffeic acid	1.759
10.953	Vanillic acid	0.286
11.653	Ferulic acid	1.323
12.539	Salicylic acid	1.154
13.087	Benzoic acid	2.306
13.404	Coumaric acid	4.251
14.948	Cinnamic acid	0.371
18.651	Chrysin	1.074

Total identified constituents		13.904

**Table 2:** Phenolics identified by RP-HPLC analysis (at *λ* = 330 nm) of the methanolic extract of *Salsola imbricata*.

Retention time	Identified constituent	Relative area %
3.867	Quercetin	0.031
11.786	Rosmarinic acid	2.734
12.025	Hesperidin	1.854
12.351	Rutin	2.101
13.195	Quercitrin	12.692
14.668	Naringenin	1.300
14.989	Hesperetin	0.730
16.153	Apigenin	0.474

Total identified constituents		21.916

**Table 3:** Effects of prolonged oral administration of the ethanolic extract of *Salsola imbricata *on the weight of the body and sexual organs of male rats.

Groups	Body weight (gm)		Testis^a^	Seminal vesicles^a^	Prostate^a^	Epididymis^a^
	Initial	Final				
Group I	208.9 ± 9.6	326.3 ± 10.3	2.31 ± 0.10	0.135 ± 0.02	0.40 ± 0.01	0.48 ± 0.01
Group II	203.2 ± 7.6	321.2 ± 13.1	2.01 ± 0.45	0.323 ± 0.09	0.37 ± 0.06	0.29 ± 0.09
Group III	207.3 ± 9.3	317.6 ± 12.3	1.83 ± 0.29	0.188 ± 0.02	0.42 ± 0.06	0.195 ± 0.06

^a^g/100 g body weight; group I: control; group II received the extract at dose of 250 mg/kg b. wt.; group III received the extract at dose of 500 mg/kg b. wt.

**Table 4:** Effect of prolonged oral administration of the ethanolic extract of *Salsola imbricata* on the cauda epididymal sperms characteristics on male rats.

Groups	Sperm counts (million/mm^3^)	Sperm motility (%)	Sperm abnormalities
Group I	50.7 ± 3.4	72.3 ± 5.1	6.4 ± 0.4
Group II	30.1 ± 2.9^*^	59.7 ± 2.4	17.6 ± 3.7^*^
Group III	25.8 ± 5.5^*^	48.4 ± 1.9^*^	30.1 ± 4.1^*^

^*^ *P* < 0.01; group I: control; group II received the extract at dose of 250 mg/kg b. wt.; group III received the extract at dose of 500 mg/kg b. wt.

**Table 5:** Effect of prolonged oral administration of the ethanolic extract of *Salsola imbricata* on the hormones level (ng/mL) on male rats.

Groups	LH	FSH	Testosterone
Group I	1.39 ± 0.12	8.07 ± 0.31	1.72 ± 0.10
Group II	1.38 ± 0.11	7.99 ± 0.34	1.81 ± 0.18
Group III	1.35 ± 0.15	8.14 ± 0.27	1.85 ± 0.14

Group I: control; group II received the extract at dose of 250 mg/kg b. wt.; group III received the extract at dose of 500 mg/kg b. wt.
